# Growing-season carbon budget of alpine meadow ecosystem in the Qinghai Lake Basin: a continued carbon sink through this century according to the Biome-BGC model

**DOI:** 10.1186/s13021-023-00244-y

**Published:** 2023-12-19

**Authors:** Meng-ya Zhang, Yu-jun Ma, Peng Chen, Fang-zhong Shi, Jun-qi Wei

**Affiliations:** 1 School of Geography and Planning, Sun Yat-Sen University, Guangzhou, 510275 China; 2 School of Natural Resources, Faculty of Geographical Science, Beijing Normal University, Beijing, 100875 China; 3 State Key Laboratory of Earth Surface Processes and Resource Ecology, Faculty of Geographical Science, Beijing Normal University, Beijing, 100875 China

**Keywords:** Carbon budget, Biome-BGC, Sensitivity analysis, Climate change, Qinghai Lake Basin

## Abstract

**Background:**

The alpine meadow is one of the most important ecosystems in the Qinghai-Tibet Plateau (QTP), and critically sensitive to climate change and human activities. Thus, it is crucial to precisely reveal the current state and predict future trends in the carbon budget of the alpine meadow ecosystem. The objective of this study was to explore the applicability of the Biome-BGC model (BBGC) in the Qinghai Lake Basin (QLB), identify the key parameters affecting the variation of net ecosystem exchange (NEE), and further predict the future trends in carbon budget in the QLB.

**Results:**

The alpine meadow mainly acted as carbon sink during the growing season. For the eco-physiological factors, the YEL (Yearday to end litterfall), YSNG (Yearday to start new growth), CLEC (Canopy light extinction coefficient), FRC:LC (New fine root C: new leaf C), SLA (Canopy average specific leaf area), C:N_leaf_ (C:N of leaves), and FLNR (Fraction of leaf N in Rubisco) were confirmed to be the top seven parameters affecting carbon budget of the alpine meadow. For the meteorological factors, the sensitivity of NEE to precipitation was greater than that to vapor pressure deficit (VPD), and it was greater to radiation than to air temperature. Moreover, the combined effect of two different meteorological factors on NEE was higher than the individual effect of each one. In the future, warming and wetting would enhance the carbon sink capacity of the alpine meadow during the growing season, but extreme warming (over 3.84 ℃) would reduce NEE (about 2.9%) in the SSP5-8.5 scenario.

**Conclusion:**

Overall, the alpine meadow ecosystem in the QLB generally performs as a carbon sink at present and in the future. It is of great significance for the achievement of the goal of carbon neutrality and the management of alpine ecosystems.

## Background

The sixth assessment report of IPCC stated that global air temperature has risen by 1.1 ℃ relative to pre-industrial levels since the nineteenth century, and it was projected to increase by 1.5 ℃ compared to pre-industrial levels over the next 20 years [1]. The Qinghai-Tibet Plateau (QTP) plays an important role in Asian climate, and is one of the most sensitive regions to recent climate change. Between 1960 and 2010, the warming rate on the QTP achieved 0.2 ℃ per decade, which is evidently higher than that in the other regions around the world [2]. On the other hand, climate change has an obvious impact on the structure and function of global ecosystems [3, 4]. Net Ecosystem Exchange (NEE) is a vital link in biogeochemical cycles and a meaningful indicator of ecosystem functional traits [5]. It refers to the variation in carbon exchange between terrestrial ecosystems and the atmosphere caused by a combination of plant photosynthesis, carbon storage in canopy air, and carbon emissions from biotic and abiotic respiration in ecosystems [6]. Therefore, NEE change has received lots of attentions in recent years.

The QTP is the highest plateau in the world, and has fragile alpine grassland ecosystems, which are highly sensitive to climate change [7]. Previous studies showed that more than 50% of grassland carbon in China was stored in the grasslands of the QTP [8]. To reveal the possible variation of this critical carbon pool, lots of researches have investigated the carbon budget under varied scenarios, including climate change (air temperature, precipitation, radiation, etc.), permafrost degradation, grazing, land utilization, and so on [9–11]. From 2002 to 2020, among the 32 eddy covariance sites across the alpine ecosystems, 26 of them were reported as carbon sink, in which the alpine marshlands had the highest net ecosystem productivity (NEP) of 104.7 ± 59.0 g C·m^−2^·a^−1^ [12]. For different seasons, alpine grasslands act as carbon sink during the growing season, and as carbon source in the non-growing season, due to the influence of vegetation growth and microbial activity [13]. In addition, the carbon source or sink function of alpine grassland exists spatially heterogeneous. For example, the alpine meadow in the northeast and southeast of QTP was primarily carbon sink with the mean annual NEE of -141.89 ± 56.98 g C·m^−2^·a^−1^, which was affected by air temperature and atmospheric CO_2_ concentration [14]. In contrast, the alpine grassland in arid and semi-arid areas presented as weak carbon sinks or carbon sources in the western QTP due to the limitation of surface soil moisture.

Global warming has extended the length of vegetation growing season, which increased about 81% of productivity in the grassland ecosystems on the QTP; however, it has also enhanced the decomposition of soil organic carbon and reduced NEP by more than 1 g C·m^−2^·a^−1^ on the southwest of QTP, thus weakening carbon sink capacity in the alpine ecosystems [15]. Mu et al. found that warming accelerated the decomposition of carbon after permafrost collapse, and transformed ecosystems from carbon sinks (1.75 μmol C·m^−2^·s^−1^) to weak carbon sources (0.05 μmol C·m^−2^·s^−1^) during the growing season on the northern QTP [16]. The Qinghai Lake Basin (QLB) located in the northeast of QTP, and is one of the most sensitive areas of global climate change [17]. As the primary vegetation type of QTP, the alpine meadow adapts to the long-term low temperature environment, and is highly sensitive to the environment change [18, 19]. Consequently, additional research is required to determine whether future climate change would strengthen or diminish the carbon sink capacity of the alpine meadow ecosystem on the QTP.

Currently, there are three techniques to assess NEE, i.e., ground-based eddy covariance (EC) flux observation [20], remote sensing inversion [21], and process-based model simulation [22]. Although the EC flux observation is relatively accurate, it can only represent the CO_2_ fluxes in a limited area, and is usually used to reveal the NEE dynamic or to evaluate model performance [23]. The remote sensing inversion can obtain large-scale carbon flux data, but is difficult to perfectly elucidate the interaction mechanism between vegetation growth and the environment conditions [24]. In contrast, the process-based model is widely used to simulate the ecosystem fluxes of carbon, nitrogen, and water [25, 26]. As one of the process-based models, the Biome-BGC (BBGC) has been utilized to explore the future trends in carbon budget and its response to the variation of eco-physiological parameters. Moreover, its applicability has been demonstrated in the Zhenqin alpine meadow, Wudaoliang alpine grassland and other areas [27, 28]. The freeze–thaw cycling has an obvious impact on vegetation phenology and hydrological process, and further influences carbon budget on the QTP [29]. By combining with remote sensing phenology theories and hydrothermal models, the simulation accuracy of the BBGC has been improved to simulate the NEE seasonal dynamics effectively [30, 31]. Qi et al. pointed out that from 2005 to 2008, the alpine meadow ecosystem on the QTP acted as weak carbon sink, and short-term warming would increase the NEE by 29.6% [32]. Liu et al. found that air temperature and precipitation were the two dominant factors controlling carbon budget of the alpine grassland on the QTP, and the interaction between these two factors would have a higher effect on NPP than the effect of each one [33]. However, most current researches have concentrated on how air temperature and precipitation affect the regional carbon budget, while how sensitive carbon flux to other hydrothermal parameters like VPD (vapor pressure deficit) and shortwave radiation is not clear. Consequently, our study utilizes the BBGC to simulate the NEE of the alpine meadow ecosystem in the QLB.

The objectives of this study were to (1) verify the applicability of the BBGC in the QLB; (2) investigate the key parameters influencing the growing-season carbon budget of the alpine meadow ecosystem; (3) predict the future trends in growing-season carbon budget of the alpine meadow under different climate change scenarios.

## Methods

### Study area and field observation

**Fig. 1:**
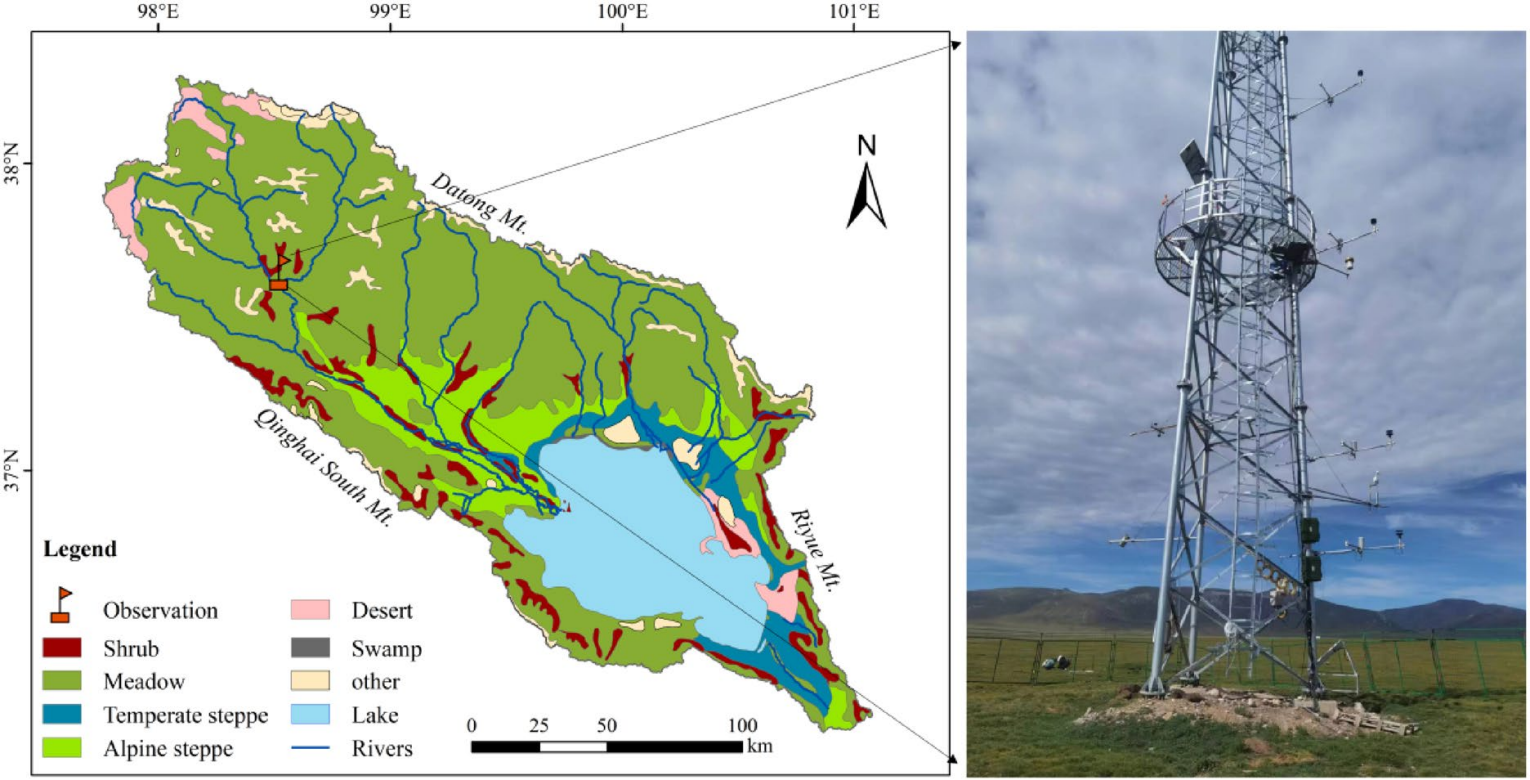
Distribution of vegetation types and the location of EC tower in the QLB

The Qinghai Lake is the largest inland saline lake in China, and located in the northeastern part of the QTP, with an altitude of 3194 m above sea level. The entire watershed is in a high altitude, cold and semiarid climate zone. The main vegetation types include temperate steppes, alpine steppes, alpine shrubs, and alpine meadows with increasing altitude (Fig. 1). The eddy covariance (EC) observatory is located in the northern part of the QLB, and the mean annual air temperature and precipitation from 2019 to 2021 were -2.17 ℃ and 418.8 mm, respectively. Approximately 70–80% of the annual precipitation occurs in the summer and early autumn. The main soil type is sandy loam, and the dominant species of the alpine meadow are *Kobresia* and *Stipa purpurea* [34].

An EC system was installed on a 40-m-high tower. The open-path infrared gas analyzer (LI-7500, LI-Cor, USA) was installed at a height of 2 m above the canopy to measure fluctuations of water vapor and carbon dioxide concentrations, while the three-dimensional sonic anemometer (WindMaster, Gill, UK) at the same height was used to measure horizontal and vertical wind velocity components (u, v, and w). All the EC data were recorded by a CR3000 data logger (Campbell Scientific Inc., USA) with half-hour interval from 2019 to 2021. Data in 2020 were missing due to equipment malfunction. A weather station (Dynamax Inc., USA) was set up to measure meteorological data such as air temperature, precipitation, four-component radiation, relative humidity, VPD, and wind speed. All the meteorological data was recorded by a CR1000 data logger (Campbell Scientific Inc., USA) with 10-min interval. More details on the in-situ instrument specification were described by Ma et al. [35].

The 10-Hz raw EC series data was processed by EdiRe software developed by the University of Edinburgh, and the correction includes spike removal, lag correction of carbon dioxide relative to the vertical wind component, sonic virtual temperature correction, the performance of the planar fit coordinate rotation, corrections for density fluctuation (WPL correction), and frequency response correction [36]. In addition to these data processing steps, both the quality control of half-hourly flux data and standardized mechanism to fill NEE gaps are needed for adequate data processing. All missing data were marked as -9999 (no data), and marginal distribution sampling (MDS) algorithm in the REddyProc gap-filling tool was used to fill gaps in the flux measurement data. More details about the data processing can be found in Wei et al. [37].

### Input data of Biome-BGC model

The driving data for the BBGC (4.2 version) include site-specific data, daily meteorological data, and eco-physiological parameters. The site-specific data include altitude, latitude, longitude, and soil texture. The daily meteorological data contains daytime average air temperature, daily maximum air temperature, minimum air temperature, precipitation, VPD, daylight average shortwave radiation and daylength. The alpine meadow was classified as C3 grass. In the BBGC, among 43 eco-physiological parameters, 31 of them were used effectively (Table 1). In this study, C:N_leaf_, C:N_roots_, and SLA were determined by filed measurement. The experimentation details can be found in Xiang et al. [38]. The other parameters were inaccessible, and mainly came from references and model default values.

**Table 1:** Eco-physiological parameters setting and acquisition ways in the BBGC

Parameters	Values	Symbol	Units	Sources
Yearday to start new growth	110	YSNG	Day of year	[25]
Yearday to end litterfall	280	YEL	Day of year	[25]
Transfer growth period as fraction of growing season	0.9	TGP	/	Optimized
Litterfall as fraction of growing season	0.46	LAFO	/	Optimized
Annual leaf and fine root turnover fraction	1.0	LFRT	1/yr	Model default value
Annual whole-plant mortality fraction	0	WPM	1/yr	Field measurement
Annual fire mortality fraction	0	FMF	1/yr	Field measurement
New fine root C: new leaf C	2.5	FRC:LC	/	Optimized
Current growth proportion	0.47	CGP	/	Optimized
C:N of leaves	18.7	C:N_leaf_	kgC/kgN	Field measurement
C:N of leaf litter, after retranslocation	65	C:N_lit_	kgC/kgN	Optimized
C:N of fine roots	77.35	C:N_root_	kgC/kgN	Field measurement
Leaf litter labile proportion	0.39	L_lab_	/	Model default value
Leaf litter cellulose proportion	0.44	L_cel_	/	Model default value
Leaf litter lignin proportion	0.17	L_lig_	/	Model default value
Fine root labile proportion	0.3	R_lab_	/	Model default value
Fine root cellulose proportion	0.45	R_cel_	/	Model default value
Fine root lignin proportion	0.25	R_lig_	/	Model default value
Canopy water interception coefficient	0.021	CWIC	1/LAI/d	Model default value
Canopy light extinction coefficient	0.37	CLEC	/	Optimized
All-sided to projected leaf area ratio	2.0	LAI_all:proj_	/	Model default value
Canopy average specific leaf area	13.24	SLA	m^2^/kgC	Field measurement
Ratio of shaded SLA:sunlit SLA	2.0	SLA_sha:sun_	/	Model default value
Fraction of leaf N in Rubisco	0.15	FLNR	/	Model default value
Maximum stomatal conductance	0.007	g_max_	m/s	[25]
Cuticular conductance	0.00001	g_cut_	m/s	Model default value
Boundary layer conductance	0.04	g_bl_	m/s	Model default value
Leaf water potential: start of conductance reduction	– 0.6	LWP_s_	Mpa	Model default value
Leaf water potential: complete conductance reduction	– 2.3	LWP_c_	Mpa	Model default value
Vapor pressure deficit: start of conductance reduction	930	VPD_s_	Pa	Model default value
Vapor pressure deficit: complete conductance reduction	4100	VPD_c_	Pa	Model default value

To establish a stable state, the model first goes through the Spin-up mode, which starts with a very low soil carbon content and repeats the meteorological data. Then, the Normal mode is used to generate the final simulation results.

### Parameter optimization

Based on the PEST model, we used the daily carbon flux data in 2019 and 2021 to optimize and fit the eco-physiological parameters in the BBGC. The core of the PEST model is the Gauss-Marquardt–Levenberg algorithm, which makes the simulation result as more uniform as the observed value (Eq. 1) [39]. The objective function is as follows:

1 $$\Phi = (\widehat{y} - y - H(\widehat{x} - x))^{T} O(\widehat{y} - y - H(\widehat{x} - x))$$

where: y is a vector with m elements (the number of measured values), x is a vector with n elements (the number of parameters), $\widehat{{\text{y}}}$ is a vector of the simulation result, $\widehat{x}$ is a parameter vector to be estimated, H is a Jacobian partial derivative matrix with m rows and n columns, T represents a transpose symbol, O is a weight matrix of measured values with m rows and m columns.

### Evaluation of model applicability

The determination coefficient (R^2^, Eq. 2) and root mean square error (RMSE, Eq. 3) were used to evaluate the applicability of the BBGC in the QBL.

2 $$R^{2} = \frac{{\sum\limits_{i = 1}^{n} {(\widehat{y}_{i}^{2} - \overline{y}_{i}^{2} )} }}{{\sum\limits_{i = 1}^{n} {(y_{i} - \overline{y}_{i}^{2} )} }}$$

3 $$RMSE = \sqrt {\frac{1}{n}\sum\limits_{i = 1}^{n} {(\widehat{y}_{i} - y_{i} )^{2} } }$$

where ${\widehat{y}}_{i}$ is the observed value, ${y}_{i}$ is the simulated value, and ${\overline{y} }_{i}$ is the average of the observed values.

### Sensitivity analysis

Previous studies showed that air temperature and precipitation are projected to increase by 2.24 ℃ and 12.8% in 2100 relative to 2020 under the SSP2-4.5 scenario, respectively [40]. Therefore, we set air temperature change between – 2.0 and 2.0 ℃ with 0.5 ℃ intervals, and precipitation, VPD, and shortwave radiation change between – 12% and 12% with 3% intervals.

In this study, there were 31 effective eco-physiological parameters in the BBGC, some of them were highly relative to others or set to zero (WPM and FMF). Therefore, only 22 eco-physiological parameters were involved in the further sensitivity analysis (Table 2), and all the change in them were set between – 12% and 12% with 3% interval to keep consistent with precipitation. For the length of growing season (the difference between YSNG and YEL with initial value 170), three scenarios were considered, i.e., (1) only change YSNG, (2) only change YEL and (3) change both YSNG and YEL.

**Table 2:** Value range of the eco-physiological parameters used in sensitivity analysis

No	Parameter symbol	Value range of parameters
1	YSNG	[97, 123]
2	YEL	[246, 314]
3	TGP	[0.8, 1.0]
4	LAFO	[0.41, 0.52]
5	FRC:LC	[2.2, 2.8]
6	CGP	[0.41, 0.52]
7	C:N_leaf_	[16.46, 20.94]
8	C:N_lit_	[57.2, 27.8]
9	C:N_root_	[68.07, 86.63]
10	CWIC	[0.018, 0.024]
11	CLEC	[0.33, 0.41]
12	LAI_all:proj_	[1.76, 2.24]
13	SLA	[11.65, 14.83]
14	SLA_sha:sun_	[1.76, 2.24]
15	FLNR	[0.13, 0.17]
16	g_max_	[0.006, 0.008]
17	g_cut_	[0.0000088, 0.000112]
18	g_bl_	[0.035, 0.045]
19	LWP_s_	[– 0.67, – 0.53]
20	LWP_c_	[– 2.576, – 2.024]
21	VPD_s_	[818, 1141]
22	VPD_c_	[3608, 4592]

The eco-physiological parameters were classified by the sensitivity discriminant index (D_sen_, Eq. 4) [41]. It was divided into three levels, with those greater than 20% being highly sensitive, those between 10 and 20% being medium sensitive, and those below 10% being insensitive [42, 43].

4 $$D_{sen} = \frac{{\sum\limits_{i = 1}^{n} {\left| {NEE_{i} - NEE_{def} } \right|} }}{{n \times NEE_{def} }} \times 100\%$$

where *NEE*_*i*_ is the NEE value simulated by the model after the ith float of the parameter, *NEE*_*def*_ is the NEE value simulated based on the localized parameter, and *n* is the number of floats of the parameter.

### Climate scenarios design

Referring to the future trends in climate change simulated by the Coupled Model Intercomparison Project Phase 6 (CMIP6) on the QTP [40], the linear trends in air temperature and precipitation were set to be 0.28 ℃ (10 a^−1^) and 1.56% (10 a^−1^) for the SSP2-4.5 scenario (medium radiation forcing), and 0.64 ℃ (10 a^−1^) and 3.8% (10 a^−1^) for the SSP5-8.5 scenario (high radiation forcing), respectively. The outputs simulated by no change in air temperature and precipitation were used as the reference.

## Results

### Seasonal variation of NEE based on EC observation

**Fig. 2:**
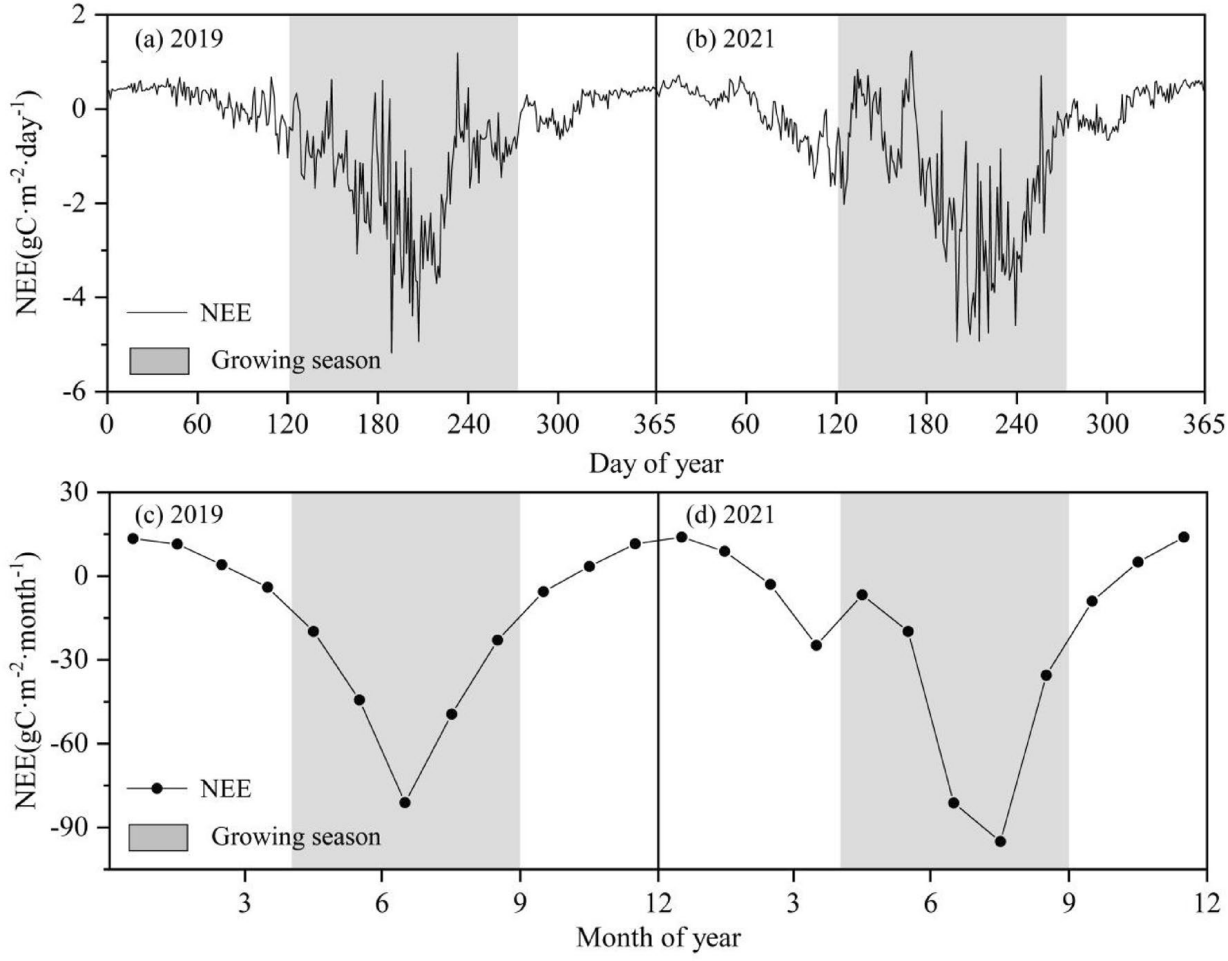
Variation of mean daily and monthly NEE in 2019 (**a**), (**c**) and 2021 (**b**), (**d**) of the alpine meadow ecosystem in the QLB

The mean annual NEE was – 207.8 g C·m^−2^ in 2019 and 2021 (Fig. 2), and the maximum CO_2_ uptake appeared in late July (– 5.17 g C·m^−2^·day^−1^ in 2019 and – 4.94 g C·m^−2^·day^−1^ in 2021) due to the favorable hydrothermal conditions. During the growing season (from May to September), the cumulative NEE was higher in 2021 (– 238.13 g C·m^−2^) than that in 2019 (– 217.46 g C·m^−2^). During the non-growing seasons, the total NEE in 2019 and 2021 were 34.77 g C·m^−2^ and 5.21 g C·m^−2^, respectively. Generally, the fluctuation of carbon fluxes in the alpine meadow was relatively low in the non-growing season.

### Simulation of NEE by Biome-BGC model

**Fig. 3:**
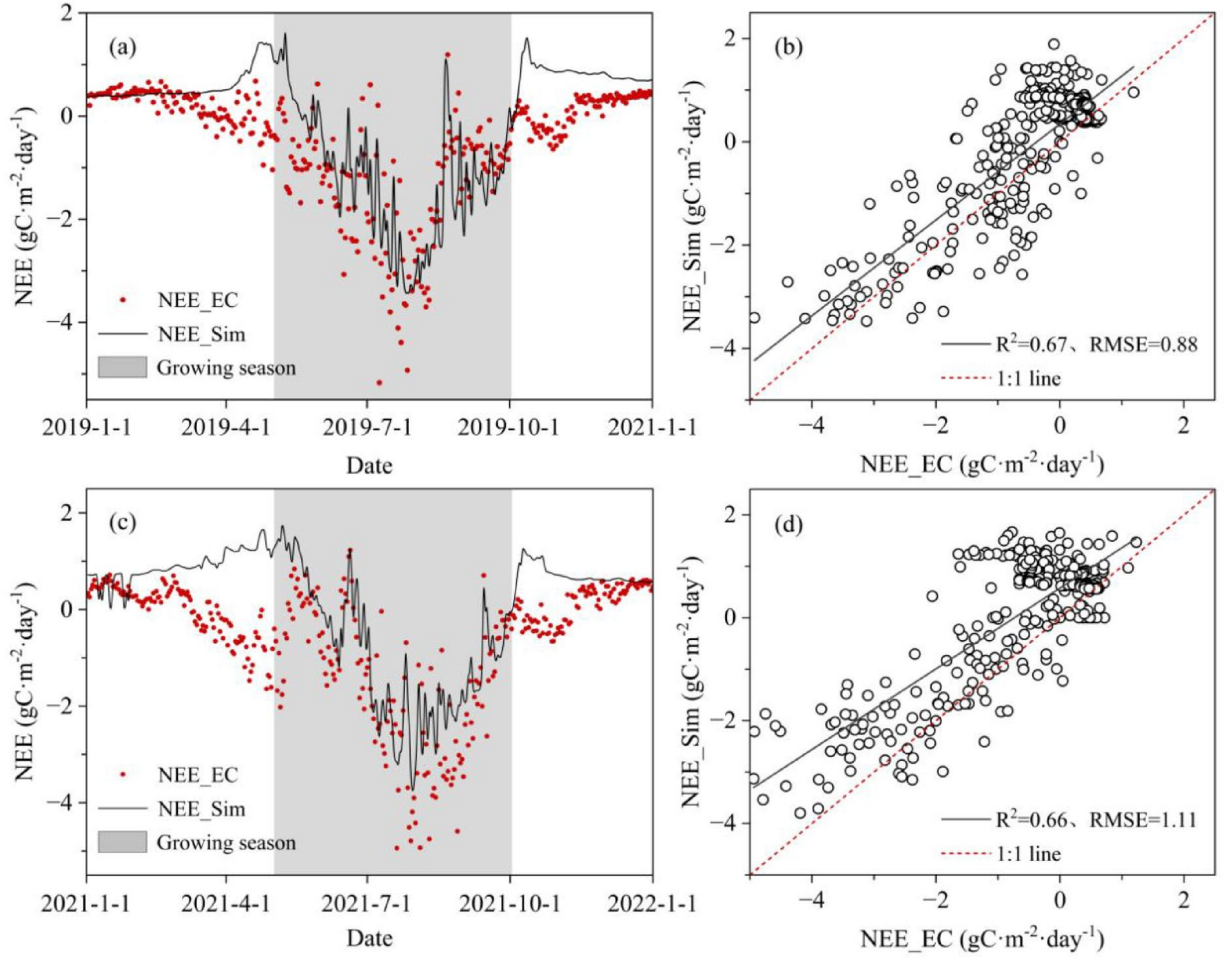
Comparison of NEE measured by EC (NEE_EC) and simulated by the BBGC (NEE_Sim). **a**, **b** were calibration results in 2019; **c**, **d** were validation results in 2021

To validate the simulation results, the measured EC flux data was compared with outcomes estimated by the local parametric model (Fig. 3). The simulation result in 2019 showed that R^2^ was 0.67 and RMSE was 0.88 g C·m^−2^·day^−1^, and the corresponding values in 2021 were 0.66 and 1.11 g C·m^−2^·day^−1^, respectively. Generally, the trend in simulated values in the growing season was more consistent with the observed values than that in the non-growing season. It indicated the BBGC had good applicability in the simulation of NEE during the growing season for the alpine meadow ecosystem in the QLB.

### Sensitivity analysis of parameters in Biome-BGC model

**Fig. 4:**
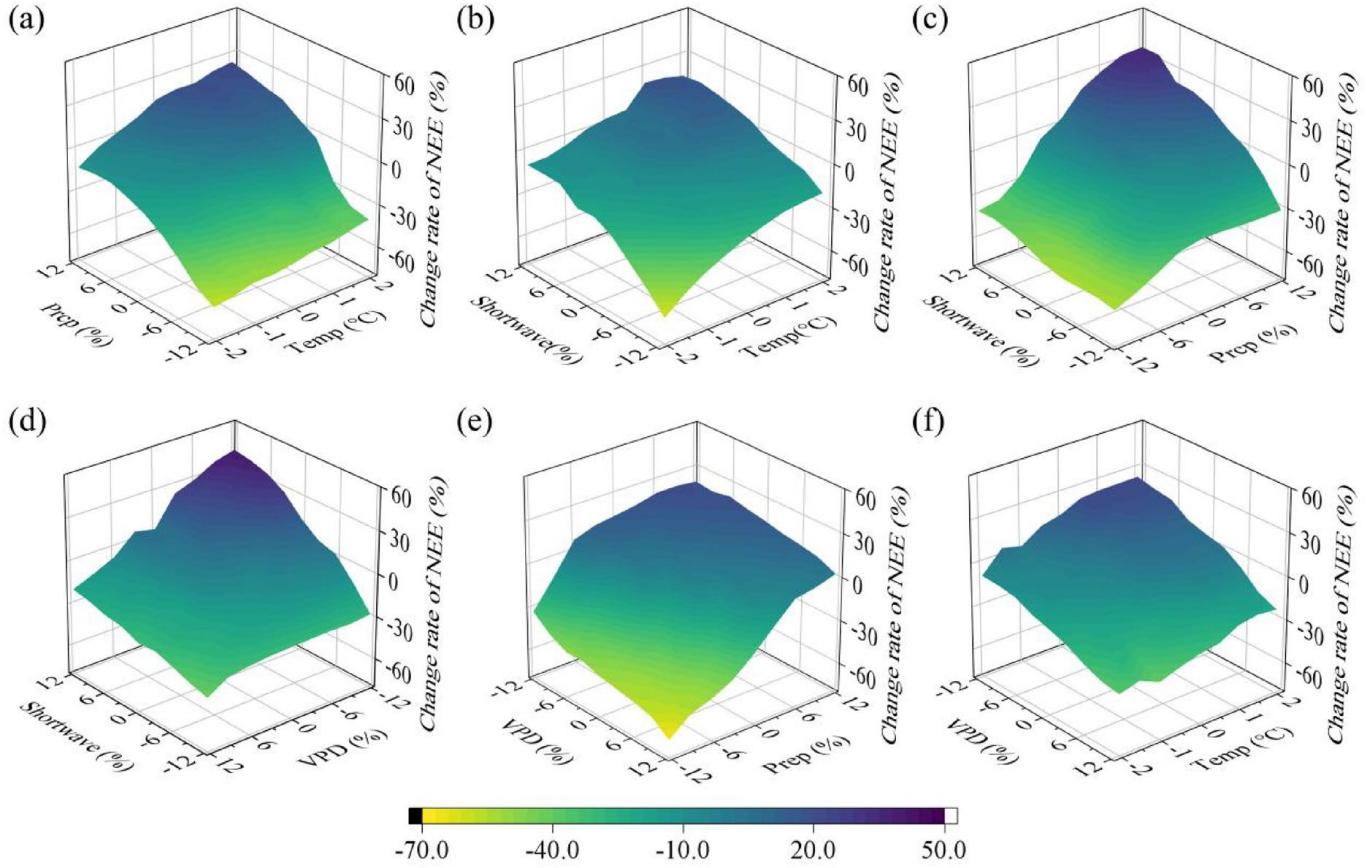
The variation of NEE under synergistic effects of different environmental factors during growing season of the alpine meadow ecosystem in the QLB. Environmental factors include air temperature (Temp), precipitation (Prcp), vapor pressure deficit (VPD) and shortwave radiation (Shortwave). **a**, **b**, **c**, **d**, **e**, and **f** represent the combined effect of Prcp and Temp, Shortwave and Temp, Shortwave and Prcp, Shortwave and VPD, VPD and Prcp, VPD and Temp on NEE, respectively

During the growing season, NEE climbed steadily by 24.08% (from – 1.04 g C·m^−2^·day^−1^ to – 1.29 g C·m^−2^·day^−1^) due to the combined effect of both 2 ℃ increase in air temperature and 12% increase in precipitation, while a strongly reduction in NEE (approximately 57%, from – 1.04 g C·m^−2^·day^−1^ to – 0.44 g C·m^−2^·day^−1^) was observed when both 2 ℃ decrease in air temperature and 12% decrease in precipitation were combined (Fig. 4a). What’s more, NEE would reduce more than 30% when precipitation dropped by 12%, regardless of the change in air temperature. Meanwhile, the combined effect of both air temperature and precipitationdecrease was greater than that of precipitation decrease separately.

Increase in both air temperature and shortwave radiation had a positive effect on NEE (Fig. 4b). However, when over 1.5 ℃ increase in air temperature and over 9% increase in shortwave radiation were combined, NEE remained unchanged or even decreased slightly relative to the combined effect of 1.5 ℃ increase in air temperature and 12% increase in shortwave radiation. Meanwhile, the influence of shortwave radiation on NEE was higher than that of air temperature. The effect of 12% decrease in shortwave radiation on the variation of NEE (30%) was higher than that (20.58%) of 2 ℃ decrease in air temperature. When both shortwave radiation and precipitation increased by 12%, NEE was evidently enhanced by 34.20% (from – 1.04 g C·m^−2^·day^−1^ to -1.396 g C·m^−2^·day^−1^), which was higher than the combined effect of air temperature and precipitation (Fig. 4c).

NEE raised strongly (from -1.04 g C·m^−2^·day^−1^ to more than -1.25 g C·m^−2^·day^−1^) when shortwave radiation increased by more than 6% and VPD reduced more than 6% at the same time, but the other combinations of their variation had a negative impact on NEE (Fig. 4d). NEE increased consistently when increased precipitation and decreased VPD were combined (Fig. 4e). However, it should be noticed that a maximum reduction of 66.75% in NEE (from -1.04 g C·m^−2^·day^−1^ to -0.335 g C·m^−2^·day^−1^) would result from the combined effect of 12% drop in precipitation and 12% increase in VPD. Meanwhile, the influence of VPD on NEE was lower than that of precipitation. The effect of 12% decrease in precipitation on the variation of NEE (49.69%) was higher that of 12% decrease in VPD (14.80%). In addition, the combined effect of 2 ℃ increase in air temperature and 12% decrease in VPD on the variation of NEE (23.25%) was much lower than that (39.32%) of 2 ℃ increase in air temperature and 12% decrease in precipitation (Fig. 4f).

**Fig. 5:**
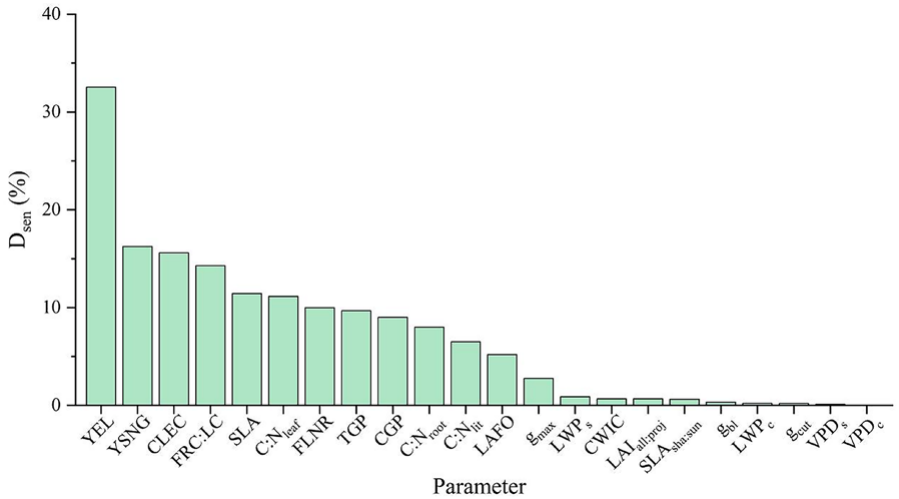
Sensitivity discriminant index (D_sen_) of eco-physiological parameters in the BBGC

Among 22 eco-physiological parameters for the sensitivity analysis (Fig. 5), the highly sensitive parameters include YEL (32.54%), YSNG (16.25%), CLEC (15.60%), FRC:LC (14.28%), SLA (11.44%), as well as C:N_leaf_ (11.14%). The parameters with high sensitivity had a greater influence on NEE, and those parameters with D_sen_ above 10% were chosen to further analyze the influence of their different amplitude on NEE. Considering that FLNR directly affects the first step of CO_2_ fixation by vegetation (carboxylation), it was also included in the further analysis [44].

**Fig. 6:**
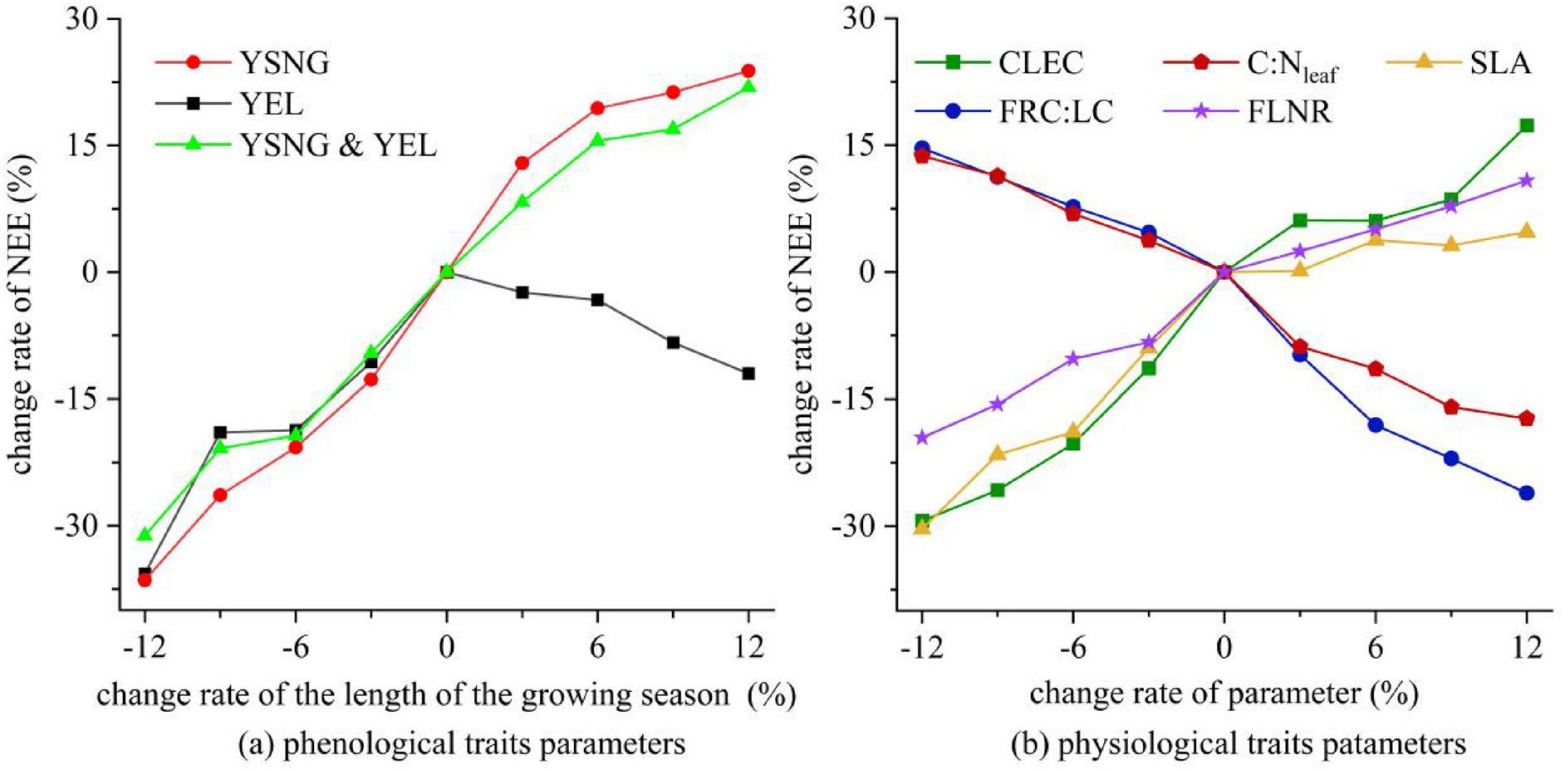
Effects of vegetation phenology traits parameters (**a)** and physiological traits parameters (**b)** on the simulation results of NEE during the growing season of alpine meadow ecosystem in the QLB. YSNG, YEL, and YSNG & YEL represent only changing YSNG, only changing YEL, and changing both YSNG and YEL equally, respectively in (**a)**

Both the delayed green-up dates (increase in YSNG) and the advanced leaf senescence dates (decrease in YEL) would shorten the length of the growing season, and further weaken the carbon sink capacity of the alpine meadow on the QTP (Fig. 6a). In contrast, an earlier green-up dates (YSNG, -20 days) contributed to the maximum increase in NEE (about 23%, from -1.04 g C·m^−2^·day^−1^ to -1.28 g C·m^−2^·day^−1^). The combined effect of advanced green-up dates (YSNG, -10 days) and delayed leaf senescence dates (YEL, + 10 days) also strongly enhanced NEE by 21.87% (from -1.04 g C·m^−2^·day^−1^ to -1.267 g C·m^−2^·day^−1^). Nevertheless, NEE fell by 12.03% (from -1.04 g C·m^−2^·day^−1^ to -0.915 g C·m^−2^·day^−1^) with delayed leaf senescence dates (YEL, + 20 days).

The influence of physiological traits parameters (FRC:LC, FLNR, C:N_leaf_, CLEC and SLA) on NEE showed that the larger variation in parameters, the more obvious variation in NEE (Fig. 6b). When these parameters varied from -12 to 0%, FRC:LC and C:N_leaf_ had a positive effect on NEE, and both of them related to the C and N components in various vegetation portions. When these parameters varied from 0 to 12%, CLEC, SLA, and FLNR mainly contributed to the increase in NEE, especially when CLEC increased by 12%, the highest increase in NEE achieved 17.34% (from – 1.04 g C·m^−2^·day^−1^ to – 1.22 g C·m^−2^·day^−1^). Among all the parameters, CLEC and SLA reflect the ability of vegetation to absorb and intercept light energy, and FLNR mainly controls the content of photosynthetic enzymes, thus affecting the rate of CO_2_ assimilation of plants [45].

### NEE response to climate change

**Fig. 7:**
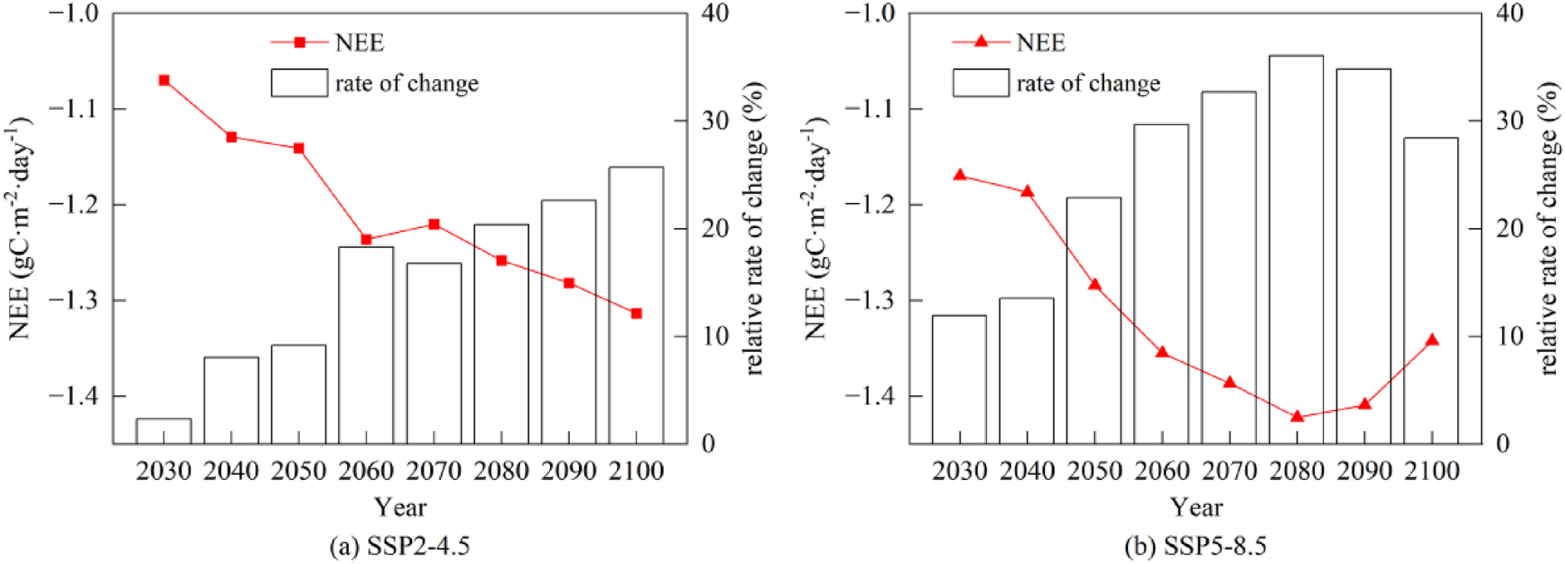
Mean daily value of NEE and its variation amplitude in different periods under different climate change scenarios

During the growing season, the response of NEE to the change in air temperature and precipitation in the alpine meadow was different under varied SSPs scenarios (Fig. 7). NEE decreased continuously in the SSP2-4.5, while first increased and then decreased in the SSP5-8.5. Between 2020 and 2060, NEE in the SSP2-4.5 was lower than that in the SSP5-8.5 with mean daily value of – 1.24 g C·m^−2^·day^−1^ (SSP2-4.5) and – 1.36 g C·m^−2^·day^−1^ (SSP5-8.5) in 2060, respectively. It indicated that from 2020 to 2060, the carbon sink capacity of the alpine meadow would be stronger in the SSP5-8.5. During 2080–2100, NEE continued to increase in the SSP2-4.5 with a substantial rise compared with the period of 2020–2060, and reached the highest value of – 1.31 g C·m^−2^·day^−1^ in 2100. In the SSP5-8.5, NEE got the highest value of – 1.42 g C·m^−2^·day^−1^ in 2080, and then showed a decreased trend from 2080 to 2100 (from – 1.42 g C·m^−2^·day^−1^ to – 1.34 g C·m^−2^·day^−1^). To clarify how air temperature and precipitation caused the variation of NEE, this study further analyzed the single effect of them on NEE under different scenarios.

**Fig. 8:**
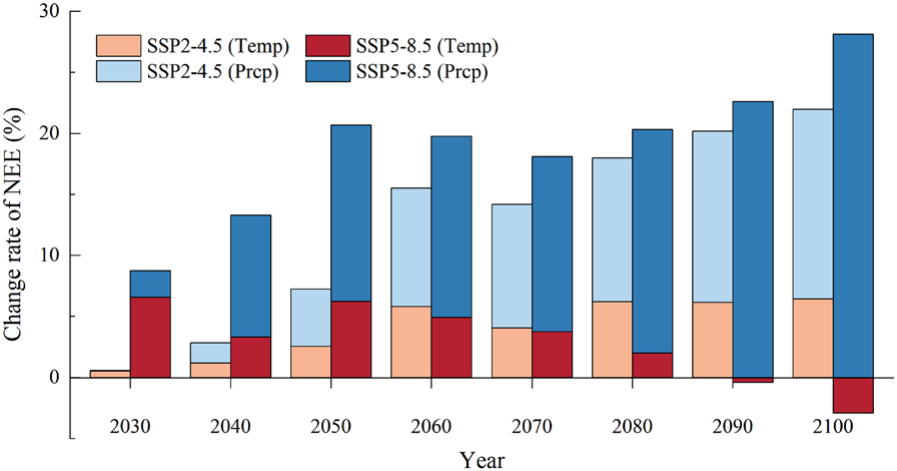
Single effect of air temperature and precipitation on the variation of NEE under different climate change scenarios during growing season of the alpine meadow ecosystem in the QLB

During 2020–2060, in the SSP2-4.5, with air temperature increased by 0.28, 0.56, 0.84, and 1.12 ℃, the NEE would increase by 0.53%, 1.19%, 2.56%, and 4.05%, respectively (Fig. 8). In contrast, with the increase in precipitation by 1.56%, 3.12%, 4.68%, and 6.24%, NEE increased by 0.06%, 1.65%, 4.69%, and 9.70%, respectively. At the same time, in the SSP5-8.5, with the increase in air temperature by 0.64, 1.28, 1.92, and 2.56 ℃, NEE raised by 6.56%, 3.31%, 6.25%, and 4.94%; with the increase in precipitation by 3.8%, 7.6%, 11.4%, and 15.2%, NEE raised by 2.19%, 9.99%, 14.43%, and 14.83%. In the SSP5-8.5, NEE decreased slightly with warming between 2030 and 2040 (from – 1.11 g C·m^−2^·day^−1^ to – 1.08 g C·m^−2^·day^−1^), and the total NEE in 2040 only increased by 1.62% compared with that in 2030, even though the NEE increased by more than 7% with increasing precipitation from 2030 to 2040 (from – 1.07 g C·m^−2^·day^−1^ to – 1.15 g C·m^−2^·day^−1^). It indicated that from 2020 to 2060, NEE was mainly controlled by air temperature in both two scenarios, and even a minor rise in air temperature and precipitation would increase the NEE and enhance the carbon sink capacity in the alpine meadow ecosystem.

In the SSP2-4.5, the further increase in air temperature could not lead to a significant rise in NEE from 2080 to 2100. In contrast, the NEE increased by 15.52% (from – 1.04 g C·m^−2^·day^−1^ to – 1.20 g C·m^−2^·day^−1^) with the increasing precipitation by 12.48%. In the SSP5-8.5, due to the increase in precipitation, the NEE continuously grew by 9.81% in 2100 compared with that in 2080. However, warming over 3.84 ℃ would result in 2.9% decrease in NEE (from – 1.04 g C·m^−2^·day^−1^ to – 1.01 g C·m^−2^·day^−1^) in 2100. It showed that the excessive warming was the primary factor weakening the carbon sink function of the alpine meadow ecosystem during the growing season.

## Discussion

### Variation characteristics of carbon budget

The mean annual NEE of the alpine meadow ecosystem in the QLB was – 227.79 g C·m^−2^·a^−1^ in 2019 and 2021. It indicated that the alpine meadow acted as a strong carbon sink, which was significantly higher than the alpine shrub (– 77.8 g C·m^−2^·a^−1^) and the alpine meadow-steppe (– 66.7 g C·m^−2^·a^−1^) [46]. The high altitude, low temperature and large temperature difference between day and night in the QLB are conducive to the carbon assimilation of vegetation [47], but also inhibit the respiration of vegetation and soil microorganisms [48]. Precipitation and temperature have the potential to influence the activities of soil microorganisms and their related enzymes, as well as improving the light use efficiency of vegetation [49]. Therefore, the favorable hydrothermal conditions in the growing season are beneficial for the accumulation of above- and below-ground biomass.

Even within the same ecosystem, the carbon source or sink function would vary widely from region to region depending on different factors. For example, the carbon source or sink function of the Lijiang alpine meadow in the southeastern of the QTP was different during the wet and dry seasons [50]. In the wet season, it performed as carbon sink (– 37.6 ± 22.5 g C·m^−2^·month^−1^), whereas it alternated between carbon source and carbon sink in the dry season. The Haibei alpine meadow played a role in weak carbon sink (– 36.2 g C·m^−2^·a^−1^) in 2015 and weak carbon source (21.6 g C·m^−2^·a^−1^) in 2016 as a result of the recurrent El Niño weather [51]. Therefore, the effects of meteorological factors have a significant impact on how ecosystems vary their capacity to operate as carbon sink or source.

As a parameter that describes temperature and relative humidity, VPD mainly affects the stomatal conductance and then affects vegetation photosynthesis [52]. In the sensitivity analysis, precipitation had a greater impact on NEE than VPD. The daily EC-observed data showed that the maximum value of VPD in the growing season reached up to 720.7 Pa in the alpine meadow. On the one hand, higher VPD may lead to a decrease in stomatal conductance of plant leaves and inhibit vegetation photosynthesis. On the other hand, the stomata are more sensitive to ambient humidity in bigger plant leaves [53, 54]. Moreover, VPD is a sensitive parameter for GPP in the BBGC based on the global sensitivity analysis, and it has a complex influence on the stomatal conductance under the conditions of sufficient light during the growing season [55]. Furthermore, radiation and temperature both have an impact on how VPD affects NEE [56]. Radiation enhancement has stronger transmission in the canopy, coupled with the reduction in VPD, which induces the opening of vegetation stomata. Thus, this process promotes photosynthesis in vegetation [57, 58].

We found that when precipitation was sufficient, the radiation had a stronger effect than air temperature on enhancing carbon sink in the alpine meadow during the growing season. The amount of radiation received by the vegetation canopy determines how much carbon dioxide could be fixed in the ecosystem [59]. Moreover, the scattering part of solar radiation can enhance the light use efficiency of vegetation canopy by interacting with vegetation leaf area index [60]. Despite the fact that photosynthesis in ecosystems is primarily powered by radiation, the canopy density of vegetation and the surrounding environment also have a significant impact on how much carbon is taken up by ecosystems [61, 62]. The carbon budget capacity of vegetation canopy under sunny conditions is less than that under cloudy conditions, and the intensive radiation in sunny conditions may inhibit photosynthesis [63, 64]. According to the Lambert–Beer formula, the BBGC first divides the leaves into two sections, shade leaf area and sunlit leaf area, and then calculates the transmission and absorption of canopy radiation by using SLA, leaf C, and the SLA_sha:sun_ [65]. SLA reflects the light-capturing ability of vegetation and is a medium-sensitive parameter in the BBGC (for D_sen_ is 22.44%). Therefore, the effect of radiation on SLA is key to simulate photosynthetic processes. However, the Lambert–Beer formula does not account for multiple scattering in vegetation media, which also increases the uncertainty of the model simulation as the radiation changes [66].

### Uncertainty of Bime-BGC in the non-growing season

One of the most recognizable features in permafrost areas on the QTP is the freeze–thaw cycle in the active layer. During this process, when the temperature decreases, soil water freezing leads to a decrease of liquid water content. On the contrary, when the temperature increases, the melting of frozen water leads to an increase in the liquid water content [67]. The difference in temperature between the soil shallow layer and the frozen layer would result in a vertical transportation in soil water [68]. Previous study also suggested that there is an obvious vertical differentiation pattern in soil water on the QTP [69]. But soil water dynamic is simplified in the BBGC, as only one soil layer is defined.

In addition, at the stage of freezing and thawing, soil water affected the physiological processes in vegetation roots, and further affect soil respiration. Meanwhile, the alternate between freezing and thawing affects soil microorganisms in complex ways, and it may induce microbes to reverse their physiological acclimation to freezing rapidly and accelerate basal respiration by up to 30% [70]. However, this model does not fully consider the variation of soil respiration caused by soil water dynamic.

### Sensitive parameters for carbon budget

In this study, we found that NEE mainly response to the eco-physiological parameters which represent vegetation phenology (YEL and YSNG), or characterize the ratio of C and N content in different parts of vegetation (FRC: LC, FLNR and C:N_leaf_), or reflect the ability of vegetation to absorb and reflect light radiation (CLEC and SLA). In previous study, Li et al. suggested that the sensitive parameters affecting NPP in *Pinus knotata* and broadleaf forests were mainly C:N_leaf_, C:N_root_, SLA, and CWIC based on the global sensitivity analysis [71]. Liu et al. pointed out that in the Hainan rubber forest, NEE was mainly sensitive to CLEC, SLA, C:N_leaf_, and FLNR [72]. Overall, the sensitive eco-physiological parameters affecting the variation of NEE in this study were basically consistent with previous studies.

But how exactly do these parameters influence the simulation results of NEE? We found that C:N_leaf_, FLNR, and SLA determine the maximum rate of carboxylation in the photosynthetic module [73, 74]. Previous studies showed that the Rubisco enzyme is the first step that directly affects the fixation of carbon dioxide by vegetation [75]. Therefore, the increase in FLNR will raise the N content in Rubisco, promote the enzyme activity, and improve vegetation photosynthesis. Compared to FLNR, the increase in C:N_leaf_ have negative impact on NEE, since nitrogen content in Rubisco enzymes would diminish with the increase in C:N_leaf_ [74]. Both CLEC and SLA reflect the light-reflecting and light-harvesting capacity of vegetation. Thus, the higher they are, the more photosynthetically active radiation the leaf received for photosynthesis [76, 77]. Furthermore, FRC:LC mainly affects the carbon emission by affecting vegetation growth [78].

In the context of warm and humid climate in the future, the extension of the growing season effectively enhances the carbon sink of the alpine meadow ecosystem [79, 80], especially there is an earlier trend in the green-up dates. However, no matter how YEL changed, its variation led to the decrease in NEE. This mainly because the variation of YEL had a little effect on the duration of NEE < 0. Essentially, with YEL increased, the dates originally defined as non-growing season would transform into growing season. Considering that the simulation of NEE in the BBGC was not well fitted with the non-growing season and no-heavy precipitation days (the precipitation on the QTP is mainly occurs in summer), more carbon uptake in the growing season was not enough to offset carbon emission in the new defined growing season [81]. Therefore, the mean daily NEE in the growing season decreased compared with the initially simulated result.

### Future trends of carbon budget

The variation of NEE in both the SSP2-4.5 and the SSP5-8.5 are mainly due to warming from 2020 to 2060. In the SSP2-4.5, the driving factor for the variation of NEE shift from air temperature to precipitation between 2080 and 2100, and the capacity of the carbon sink keeps rising. While in the SSP5-8.5, NEE is still driven primarily by air temperature, and the carbon sink capacity may even decrease due to excessive warming. Because of the high altitude and low temperature, the activation of soil microbial enzymes is restricted and the mineralization process of organic matter components is slowed on the QTP [82]. Global warming has effectively alleviated this limitation, promotes the microbial metabolism in the alpine meadows, and accelerates carbon cycling during the growing season [83]. A series of warming experiments demonstrated that the variation of surface soil temperature has a great influence on NEE duo to warming. For example, during 2014–2016, warming caused the average daytime NEE decreased by 16.5% ~ 21.3% in the Heihe Basin [84]. However, Du et al. demonstrated that less than 2 ℃ increase in temperature would not have much influence on the amount of CO_2_ absorption in the ecosystem, while over 2.6 ℃ increase in temperature would change the CO_2_ absorption into CO_2_ release, and 4.8 ℃ increase in temperature would induce a large amount of CO_2_ release from the alpine meadow ecosystem (about 166.8 gC·m^−2^·year^−1^) [85]. This means that excessive warming like SSP5-8.5 would jeopardize future carbon sink capacity at the alpine meadow ecosystem on the QTP.

As water is a key factor influencing plant growth, the response of NEE to precipitation is largely dependent on the increase in soil water content after precipitation, as well as changes in above-ground net primary productivity [86, 87]. In arid and semi-arid regions, excessive warming may lead to a decrease in the available soil moisture content for vegetation [88], so that the effectiveness of precipitation was greater than that of air temperature over the long-term period of climate change in the SSP2-4.5. On the other hand, precipitation is the main variable controlling productivity and carbon sequestration in arid and semi-arid regions. The increase in precipitation regulates soil water content, makes the soil environment moister, which improving the supply of vegetation nutrients, promoting plant photosynthesis, and further accelerating carbon sequestration [89, 90]. Therefore, the continuous increase in precipitation has consistently shown a positive effect on the carbon sink capacity of the alpine meadow and mitigated the negative impact of excessive warming on NEE during the growing season.

## Conclusions

The research investigated the applicability of the BBGC, the sensitive parameters affecting the variation of NEE, and the future trend in carbon budget of the alpine meadow ecosystem during the growing season in the QLB. The main conclusions are as follows:

1. The BBGC could simulate the NEE well during the growing season, and the simulation results were not accurate during the non-growing season, which may be caused by the soil water dynamics and the increased soil respiration during the freeze–thaw cycle.
2. Among the meteorological parameters, NEE was more sensitive to precipitation than to VPD, and more sensitive to radiation than to air temperature. The eco-physiological parameters affecting NEE included phenology parameters (YEL and YSNG) and physiological traits parameters (CLEC, FRC:LC, SLA, C:N_leaf_, and FLNR), which represent the ability of vegetation to absorb and reflect light radiation and the C and N content in different parts of vegetation.
3. From 2020 to 2060, NEE would increase with warming and increasing precipitation in both SSP2-4.5 and SSP5-8.5. However, over 3.84 ℃ warming would jeopardize the carbon sink capacity of the alpine meadow in the QLB during the growing season. In the SSP2-4.5, the main factor that affect the NEE was warming during 2020–2060 and increasing precipitation between 2080 and 2100, respectively. By contrary, in the SSP5-8.5, warming was always the primary factor affecting the variation of NEE.

## Data Availability

Primary data used in this paper are available from the authors upon request (mayujun3@mail.sysu.edu.cn).
